# Symbolic Coloured SCC Decomposition

**DOI:** 10.1007/978-3-030-72013-1_4

**Published:** 2021-02-26

**Authors:** Nikola Beneš, Luboš Brim, Samuel Pastva, David Šafránek

**Affiliations:** 8 Eindhoven University of Technology, Eindhoven, The Netherlands; 9 Aalborg University, Aalborg East, Denmark; Faculty of Informatics, Masaryk University, Brno, Czech Republic

**Keywords:** strongly connected components, symbolic algorithm, edge-coloured digraphs, systems biology

## Abstract

Problems arising in many scientific disciplines are often modelled using edge-coloured directed graphs. These can be enormous in the number of both vertices and colours. Given such a graph, the original problem frequently translates to the detection of the graph’s strongly connected components, which is challenging at this scale.

We propose a new, symbolic algorithm that computes all the monochromatic strongly connected components of an edge-coloured graph. In the worst case, the algorithm performs $O(p\cdot n\cdot \log n)$ symbolic steps, where *p* is the number of colours and *n* the number of vertices. We evaluate the algorithm using an experimental implementation based on Binary Decision Diagrams (BDDs) and large (up to $2^{48}$) coloured graphs produced by models appearing in systems biology.

### 1 Introduction

Processing massive data sets poses a series of interesting computational challenges. A variety of these data sets can be modelled as very large multigraphs, augmented by a specific collection of application-dependent edge attributes. These attributes are often represented as colours and the resulting formalism is called an *edge-coloured graph* [4, 10]. Geographic information systems, telecommunications traffic, or internet data are prime examples of data that are best represented as such edge-coloured graphs. For instance, in social networking, it is typically used to identify groups of nodes related to each other by some specific criteria (Sports, Health, Technology, Religion, etc.) represented as colours. Our interest in processing huge edge-coloured graphs is primarily motivated by applications taken from systems biology [5, 29] and genetics [25] where we have to deal not only with giant graphs as measured by the number of vertices and edges but also with large sets of colours. The colours in such graphs represent various parameters that influence the dynamics of a biological system [5, 9, 46].

Fundamental graph algorithms such as breadth-first search, spanning tree construction, shortest paths, decomposition into strongly connected components

⋆ Supported by the Czech Science Foundation grant No. 18-00178S.

(SCCs), etc., are building blocks of many practical applications. For the edge-coloured graphs, the primary research focus so far has been on some of the "classical" coloured graph problems, like the determination of the chromatic index, finding sub-graphs with a specified colour property (the coloured version of the k-linked problem), properly edge-coloured cycles and paths, alternating cycles, rainbow cliques, monochromatic cliques, monochromatic cycles, etc. [1–4, 55, 33].

To the best of our knowledge, we are not aware of any work on SCC decomposition for edge-coloured graphs, even though this problem has many important applications. For example, in biological systems, connected components represent the attractors of the system. These play an essential role in determining the system's properties, since they may correspond, for example, to the specific phenotypes of a cell [21]. The parameters (e.g. reaction rates) in such systems might be represented as edge colours in the state transition graph. The knowledge of attractors and how their structure depends on parameters is vital for understanding various biological phenomena [24, 38]. Other applications where investigation of attractors is crucial include predictions of the global climate change [52], or predictions of spreading of infectious diseases such as COVID-19 [39].

There is a serious computational problem related to the processing of massive edge-coloured graphs, even the non-coloured ones, that significantly affects the tractability of SCC decomposition. The graphs often cannot be handled with standard (explicit) representations since they are too large to be kept in the main memory. Various approaches have been considered to deal with such giant graphs: distributed-memory structures, structures for representing graphs symbolically, or storing the graphs in external memory. We review these approaches in more detail in the related work section.

In [6, 13] we have initially attacked the SCC decomposition problem for massive edge-coloured graphs by developing a parallel semi-symbolic algorithm for detecting terminal SCCs. The algorithm uses symbolic structures to represent sets of parameters, while the graph itself is represented explicitly. The results have shown that the parallel semi-symbolic algorithm is not sufficient for the practical needs to tackle large graphs representing real-world problems. Those findings have motivated us to propose an entirely symbolic approach.

In this paper, we consider *edge-coloured multi-digraphs*, i.e., multi-digraphs such that each directed edge has a colour and no two parallel (i.e., joining the same pair of vertices) edges have the same colour. Here, we refer to such graphs simply as *coloured graphs*. For coloured graphs, we can define several notions of strongly connected components involving colours. We consider the simplest case, where the SCCs are *monochromatic*, that is all their edges have the same colour [35]. This choice is motivated by the application in systems biology, as mentioned above.

We propose a novel fully symbolic algorithm for detecting *all monochromatic components* in coloured graphs which is in practice significantly faster than is achievable with a naïve execution of an algorithm for symbolic SCC decomposition scanning all colours one-by-one, in particular on massive coloured graphs. This is because in many applications, the edges are largely shared among individual colours [5] and our algorithm is capable of exploiting this fact. The algorithm conceptually follows the *lock-step* reachability approach by Bloem [14] for monochromatic digraphs. The key new ingredients behind our algorithm are a careful orchestration of the forward and backward reachability for different colours and a sophisticated selection of a set of pivots.

#### 1.1 Related Work

The detection of SCCs in (monochromatic) digraphs is a well-known problem computable in linear time. Best serial (explicit) algorithms are Kosaraju-Sharir [50] and Tarjan [53], which are both inherently based on depth-first search. However, these algorithms do not scale for large graphs, e.g., those encountered in model-checking. Therefore, alternative approaches to SCC decomposition have been proposed (I/O efficient, parallel, symbolic algorithms).

The algorithm of Jiang [32] gives an I/O-efficient alternative based on a combination of depth-first and breadth-first search.

Efficient parallel distributed-memory algorithms avoid the inherently sequential DFS step [45] in several different ways. The Forward-Backward algorithm [26] employs a divide-and-conquer approach relying on picking a pivot state and splitting the graph in three independent (no crossing SCCs) parts. The approach of Orzan [44] uses a different distribution scheme called a colouring transformation employing a set of prioritised colours to split the graph into many parts at once. The recursive OWCTY-Backward-Forward (OBF) approach is proposed in [8]. It recursively splits the graph in a number of independent sub-graphs called OBF slices and applies to each slice the One-Way-Catch-Them-Young (OWCTY) technique. In [51] the authors utilise variants of Forward-Backward and Orzan's algorithms for optimal execution on shared-memory multi-core platforms. Finally, Bloemen et al. [15] utilise the important ability of Tarjan's algorithm to return detected SCCs on-the-fly. In particular, they present an on-the-fly parallel algorithm showing promising speedups for large graphs containing large SCCs. On another end, GPU-accelerated approaches to computing SCCs have been addressed, e.g., in [7, 30, 37, 56].

Computing SCCs of (monochromatic) digraphs symbolically is another way to handle giant graphs and has been thoroughly explored in literature. As in the case of efficient parallelisation, depth-first search is not feasible in the symbolic framework [28]. In consequence, many DFS-based algorithms cannot be easily revised to work with symbolically represented graphs. An algorithm based on forward and backward reachability performing $\mathcal{O}(n^2)$ symbolic steps was presented by Xie and Beerel in [57]. Bloem et al. present an improved $\mathcal{O}(n \cdot \log n)$ algorithm in [14]. Finally, an $\mathcal{O}(n)$ algorithm was presented by Gentilini et al. in [27, 28]. This bound has been proved to be tight in [20]. In [20], the authors argue that the algorithm from [27] is optimal even when considering more fine-grained complexity criteria, like the diameter of the graph and the diameter of the individual components. Ciardo et al. [59] use the idea of saturation [22] to speed up state exploration when computing each SCC in the Xie-Beerel algorithm, and compute the transitive closure of the transition relation using a novel algorithm based on saturation. Besides these generic algorithms, there have been recently also proposed symbolic SCC decomposition methods to deal with specific large graphs, e.g., graphs generated by Boolean networks [42, 58].

## 2 Problem Definition

As we have already stated in the introductory section, the SCC decomposition problem for edge-coloured graphs has remained mostly unexplored until now. We thus start this paper by introducing and formalising the notion of *coloured SCC decomposition* itself and state some of its basic properties.

Before giving exact definitions, it might be instructive to discuss the substance of the coloured SCC decomposition intuitively. There are several ways of capturing the notion of a "coloured connected component". For example, one of them is that of a colour-connectivity first introduced by Saad [47]. It is based on alternating paths in which successive edges differ in colour. However, there is no unique, universally acceptable notion of a coloured component.

In the biological application we have in mind, we want to identify a coloured component as a coloured collection of SCCs—a collection where for every colour there is a set of all relevant monochromatic SCCs. Such setting leads us to represent SCCs in the form of a relation. To that end, we first introduce such a relation for monochromatic graphs (Section 2.1) and consequently extend it to edge-coloured graphs (Section 2.2). The relation-based approach gives us also the advantage of allowing a feasible symbolic encoding of the problem.

### 2.1 Graphs and Strongly Connected Components

Let us first recall the standard definitions of a directed graph and its strongly connected components:

**Definition 1.** *A* directed graph *is a tuple $G = (V, E)$ where $V$ is a set of graph* vertices *and $E \subseteq V \times V$ is a set of graph* edges*.*

We are going to use the word *graph* to mean *directed graph* in the following. We write $u \rightarrow v$ when $(u, v) \in E$ and $u \rightarrow^* v$ when $(u, v) \in E^*$, the reflexive and transitive closure of $E$. We say that $v$ is *reachable* from $u$ if $u \rightarrow^* v$. The reachability relation allows us to decompose a graph into strongly connected components, defined as follows:

**Definition 2.** *In a graph $G = (V, E)$, a* strongly connected component *(SCC) is a maximal set $W \subseteq V$ such that for all $u, v \in W$, $u \rightarrow^* v$ and $v \rightarrow^* u$. For a fixed $v \in V$, we write $SCC(G, v)$ to denote the SCC of $G$ that contains $v$.*

If the graph $G$ is clear from the context, we can simply write $SCC(v)$. A set of vertices $S \subseteq V$ is said to be *SCC-closed* if every SCC $W$ is either fully contained inside $S$ ($W \subseteq S$), or in its complement ($W \subseteq V \setminus S$). Notice that given a vertex $v$, the set of all vertices reachable from $v$, as well as the set of all vertices that can reach $v$, are both SCC-closed.

A pivotal problem in computer science is to find the SCC decomposition of $G$. As mentioned above, we represent the decomposition in the form of an *equivalence relation* $R_{scc}$ such that the individual SCCs are exactly the equivalence classes of $R_{scc}$. The relation-based formulation of the SCC decomposition problem is the following:

**Problem 1 (SCC decomposition)** *Given a graph* $G = (V, E)$*, find the* SCC decomposition relation $R_{scc} \subseteq V \times V$ *such that* $(u, v) \in R_{scc}$ *if and only if* $SCC(u) = SCC(v)$.

Note that $SCC(u)$ is the section of the first attribute of $R_{scc}$, i.e. $SCC(u) = \{u \mid (u, v) \in R_{scc}\}$. We denote such a section in the following way: $SCC(u) = R_{scc}(u, \_)$. Here, $u$ is the specific value of an attribute at which the section is taken, and $\_$ is used in place of the attributes that remain unchanged. Such notation naturally extends to relations of arbitrary arity.

#### 2.2 Coloured SCC Decomposition Problem

We now lift the formal framework to the coloured setting. An edge-coloured graph can be seen as a succinct representation of several different graphs, all sharing the same set of vertices. Note that to emphasise the difference from the standard graphs as given in Definition 1, we sometimes call the standard graphs *monochromatic.*

**Definition 3.** *An* edge-coloured directed multi-graph *(coloured graph for short) is a tuple* $\mathfrak{G} = (V, C, E)$ *where* $V$ *is a set of vertices,* $C$ *is a set of colours and* $E \subseteq V \times C \times V$ *is a coloured edge relation.*

We also write $u \xrightarrow{c} v$ whenever $(u, c, v) \in E$. By fixing a colour $c \in C$ and keeping only the $c$-coloured edges (with the colour attribute removed), we obtain a monochromatic graph $\mathfrak{G}(c) = (V, \{(u, v) \mid (u, c, v) \in E\})$. We call this graph the *monochromatisation of* $\mathfrak{G}$ *with respect to* $c$. Intuitively, one can view the elements of $C$ as a type of graph parametrisation where the edge structure of the graph changes based on the specific $c \in C$.

The SCC decomposition relation $R_{scc}$ is extended to the coloured SCC decomposition relation $\mathfrak{R}_{scc}$ by relating every colour $c \in C$ with all SCCs of the monochromatisation $\mathfrak{G}(c)$. In consequence, the SCC decomposition problem is then lifted to the coloured SCC decomposition problem as follows:

**Problem 2 (Coloured SCC decomposition)** *Given a coloured graph* $\mathfrak{G} = (V, C, E)$*, find the* coloured SCC decomposition relation $\mathfrak{R}_{scc} \subseteq V \times C \times V$ *satisfying* $(u, c, v) \in \mathfrak{R}_{scc}$ *if and only if* $(u, v) \in R_{scc}$ *of* $\mathfrak{G}(c)$.

From this definition, we can immediately observe the following properties about the relationship of $\mathfrak{R}_{scc}$ with the terms which we have defined before:

- $R_{scc}$ of a monochromatisation $\mathfrak{G}(c)$ is exactly the section $\mathfrak{R}_{scc}(\_, c, \_)$;
- $SCC(\mathfrak{G}(c), v)$ is exactly the section $\mathfrak{R}_{scc}(v, c, \_)$.

From this, it should be immediately clear that $\mathfrak{R}_{scc}$ contains all components of the underlying monochromatisations.

## 3 Algorithm

Conceptually, our algorithm follows the *lock-step* reachability approach by Bloem [14] for monochromatic graphs. The lock-step algorithm itself is based on the basic forward-backward decomposition algorithm [57]. In this section, we first briefly introduce these two algorithms in order to explain better the key ideas behind our approach and, in particular, to explain what were the main difficulties encountered in employing the concepts of these algorithms to edge-coloured graphs. Although the algorithms were originally presented as producing a set of SCCs, we reformulate them slightly using the equivalent relation-based approach as explained in the previous section. After that, we present the coloured SCC decomposition algorithm. However, before we dive into the algorithmics, let us first briefly discuss the computation model we are using.

### 3.1 Symbolic Computation Model

As a complexity measure of our algorithm, we consider the number of symbolic steps, or more specifically, symbolic set and relation operations that the algorithm performs. As is customary, we assume that sets of vertices ($V$) and colours ($C$) can be represented symbolically (for example, using reduced ordered binary decision diagrams [17]) as well as any relations over these sets. In particular, we often talk about *coloured vertex sets*, by which we mean the subsets of $V \times C$.

Aside from normal set operations (union, intersection, difference, product and element selection), we also require some basic relational operations, all of which we outline in Fig. 1. These extra operations tend to appear in other applications as well (such as symbolic model checking [18]), and are thus typically already available in mature symbolic computation packages.

Finally, there are several derived operators that are partially specific to our application to coloured graphs. However, these can be constructed using standard set and relation operations. The intuitive meaning of the derived operators is as follows: Colours returns all the colours that appear in the given coloured vertex set. Pre and Post compute the pre and post-image of a (monochromatic or coloured) set of vertices, i.e. the set of successors or predecessors of all the vertices in the given set, respectively. Finally, Join takes a coloured vertex set $A$ and computes the set $\{(u, c, v) \mid (u, c) \in A, (v, c) \in A\}$.

### 3.2 Forward-backward Algorithm

To symbolically compute the SCCs of a graph $G = (V, E)$, Xie and Beerel [57] observed that for any vertex $v \in V$, the intersection $W = F \cap B$ of the forward reachable vertices $F = \{v' \in V \mid v \to^* v'\}$ and the backward reachable vertices $B = \{v' \in V \mid v' \to^* v\}$ is exactly the strongly connected component of $G$ which contains $v$.

The algorithm thus picks an arbitrary *pivot* $v \in V$, and divides the vertices of the graph into four disjoint sets: $W$, $F \setminus W$, $B \setminus W$ and $V \setminus (F \cup B)$. This is illustrated graphically in Fig. 2 (left). The set $W$ is then immediately reported as an SCC

| Standard set operations | | |
|---|---|---|
| pick element | $\textsc{Pick}(A)$ | arbitrary $x \in A$ |
| union | $A \cup B$ | $\{x \mid x \in A \vee x \in B\}$ |
| intersection | $A \cap B$ | $\{x \mid x \in A \wedge x \in B\}$ |
| difference | $A \setminus B$ | $\{x \mid x \in A \wedge x \notin B\}$ |
| product | $A \times B$ | $\{(x, y) \mid x \in A \wedge y \in B\}$ |
| Relation manipulation ($R \subseteq S_1 \times \ldots \times S_n$) | | |
| $i$-th section at $x$ | $\sigma_i(x, R)$ | $\{(y_1, \ldots, y_{i-1}, y_{i+1}, \ldots, y_n) \mid (y_1, \ldots, y_{i-1}, x, y_{i+1}, \ldots, y_n) \in R\}$ |
| existential quantification of the $i$-th element | $\exists_i(R)$ | $\bigcup_{x \in S_i} \sigma_i(x, R)$ |
| swap | $\textsc{Swap}(R \subseteq A \times B)$ | $\{(y, x) \in B \times A \mid (x, y) \in R\}$ |
| Derived operations ($G = (V, E), \mathfrak{G} = (V, C, E)$) | | |
| colours | $\textsc{Colours}(A \subseteq V \times C)$ | $\exists_1(A)$ |
| pre-image | $\textsc{Pre}(G, A \subseteq V)$ | $\exists_2((V \times A) \cap E)$ |
| post-image | $\textsc{Post}(G, A \subseteq V)$ | $\exists_1((A \times V) \cap E)$ |
| coloured pre-image | $\textsc{Pre}(\mathfrak{G}, A \subseteq V \times C)$ | $\exists_3((V \times \textsc{Swap}(A)) \cap E)$ |
| coloured post-image | $\textsc{Post}(\mathfrak{G}, A \subseteq V \times C)$ | $\textsc{Swap}(\exists_1((A \times V) \cap E))$ |
| coloured join | $\textsc{Join}(A \subseteq V \times C)$ | $(V \times \textsc{Swap}(A)) \cap (A \times V)$ |

**Fig. 1.** Summary of symbolic operations that appear in the presented algorithms. The derived operations can be implemented using the standard and relational operations. However, typically they also have a slightly more efficient direct implementations.

of the graph, and added into the component relation: $R_{scc} \leftarrow R_{scc} \cup (W \times W)$. It is easy to see that every other SCC is fully contained within one of the three remaining sets (they are SCC-closed), and the algorithm thus recursively repeats this process independently in each set.

The correctness of the algorithm follows from the initial observation and the fact that every vertex eventually appears in $W$ (either as a pivot or as a result of $F \cap B$). In the worst case, the algorithm performs $O(|V|^2)$ symbolic steps, since every vertex is picked as a pivot at most once and the computation of $F$ and $B$ requires at most $O(|V|)$ Pre/Post operations.

#### 3.3 Lock-step Algorithm

To improve the efficiency of the forward-backward algorithm, the lock-step approach [14] uses another important observation: To compute $W$, it is not necessary to fully compute both $F$ and $B$; only the smaller (in terms of diameter) of the two sets needs to be entirely known. With this observation, the computation of $F$ and $B$ can be modified in the following way: Instead of computing $F$ and $B$ one after the other, the computation is *interleaved* in a step-by-step manner (dovetailing). When one of the sets is fully computed, the computation of the second set is stopped. Let us call the computed set *converged* and denote it by

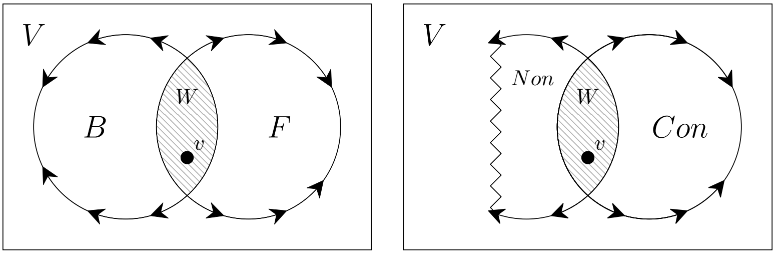

**Fig. 2.** Illustration of the difference between the forward-backward algorithm (left) and the lock-step algorithm (right). On the left, we fully compute both backward ($B$) and forward ($F$) reachable sets from the pivot $v$, identifying $W$ as $F \cap B$. On the right, without loss of generality, assume $F$ is fully computed first. It thus becomes converged ($Con$) and the computation of $B$ ($Non$) is stopped before it is fully explored.

$Con$, and the unfinished set *non-converged* and denote it by $Non$. This situation is illustrated in Fig. 2 (right).

However, even when $Con$ is fully known, we still need to finish the computation of states in $Non$ that are inside $Con$ to discover the whole component $W$. This is necessary if there are vertices $w$ in $W$ whose forward distance from $v$ (i.e. the length of the path $v \to^* w$) is short while their backward distance (the length of the path $w \to^* v$) is long, or vice versa. Such vertices are thus only discovered in one of the two reachability procedures and still need to be discovered by the other one to identify the whole component. However, an important observation is that only the vertices already inside $Con$ need to be considered in this step.

After this, the SCC can be identified and reported just as in the forward-backward algorithm. Finally, the recursion now continues in sets $Con \setminus W$ and $V \setminus Con$. This is due to $Non$ being not fully computed; we cannot guarantee that no SCC overlaps outside of $Non$ ($Non$ is not necessarily SCC-closed).

The algorithm is still correct because every vertex is eventually either picked as a pivot or discovered in some $W$. Furthermore, due to the way $Con$ and $Non$ are computed guarantees that $W$ is still a whole SCC. In terms of complexity, the algorithm performs $O(|V| \cdot \log |V|)$ symbolic steps in the worst case. To see why this is true, we may observe that every vertex appears in $W$ exactly once, and that the smaller of the two sets $Con \setminus W$ and $V \setminus Con$, let us call it $S$, is always smaller than $\frac{|V|}{2}$. The authors then argue that the price of every iteration can be attributed (up to a multiplicative constant) to the vertices in $S \cup W$ and that every vertex appears in $S$ at most $O(\log |V|)$-times.

#### 3.4 Coloured Lock-step Algorithm

When developing an algorithm for coloured graphs, we had to deal with multiple challenges which do not appear for monochromatic graphs and require careful consideration. In the following, we refer to the pseudocode in Algorithm 1.

An important observation is that the structure of components in the graph can change arbitrarily with respect to the graph colours. In consequence, our algorithm

**Algorithm 1:** Symbolic Coloured SCC Decomposition

**Function** ColouredSCC($\mathfrak{G} = (V, C, E)$)
  $\mathfrak{R}_{scc} \subseteq (V \times C \times V) \leftarrow \emptyset$;
  Decomposition($\mathfrak{G}, \mathfrak{R}_{scc}, V \times C$);
  **return** $\mathfrak{R}_{scc}$;

**Function** Decomposition($\mathfrak{G} = (V, C, E), \mathfrak{R}_{scc} \subseteq (V \times C \times V), \mathcal{V} \subseteq (V \times C)$)
  **if** $\mathcal{V} = \emptyset$ **then return**;
  $\mathcal{F}, \mathcal{B}, \overrightarrow{\mathcal{F}}, \overrightarrow{\mathcal{B}} \subseteq (V \times C) \leftarrow$ Pivots($\mathcal{V}$);
  $\overrightarrow{\mathcal{F}_u}, \overrightarrow{\mathcal{B}_u} \subseteq (V \times C) \leftarrow \emptyset$;
  $F_{lock}, B_{lock} \subseteq C \leftarrow \emptyset$;
  **while** $F_{lock} \cup B_{lock} \subset$ Colours($\mathcal{V}$) **do**
    $\overrightarrow{\mathcal{F}} \subseteq V \times C \leftarrow ($Post$(\mathfrak{G}, \overrightarrow{\mathcal{F}}) \cap \mathcal{V}) \setminus \mathcal{F}$;
    $\overrightarrow{\mathcal{B}} \subseteq V \times C \leftarrow ($Pre$(\mathfrak{G}, \overrightarrow{\mathcal{B}}) \cap \mathcal{V}) \setminus \mathcal{B}$;
    $F_{lock} \leftarrow F_{lock} \cup ($Colours$(\mathcal{V}) \setminus$ Colours$(\overrightarrow{\mathcal{F}}))$;
    $B_{lock} \leftarrow B_{lock} \cup ($Colours$(\mathcal{V}) \setminus$ Colours$(\overrightarrow{\mathcal{B}}) \setminus F_{lock})$;
    $\overrightarrow{\mathcal{F}_u} \leftarrow \overrightarrow{\mathcal{F}_u} \cup (\mathcal{F} \cap (V \times B_{lock}))$;
    $\overrightarrow{\mathcal{B}_u} \leftarrow \overrightarrow{\mathcal{B}_u} \cup (\mathcal{B} \cap (V \times F_{lock}))$;
    $\overrightarrow{\mathcal{F}} \leftarrow \overrightarrow{\mathcal{F}} \setminus (V \times B_{lock})$;
    $\overrightarrow{\mathcal{B}} \leftarrow \overrightarrow{\mathcal{B}} \setminus (V \times F_{lock})$;
    $\mathcal{F} \leftarrow \mathcal{F} \cup \overrightarrow{\mathcal{F}}$;
    $\mathcal{B} \leftarrow \mathcal{B} \cup \overrightarrow{\mathcal{B}}$;
  **end**
  $\mathcal{C}on \subseteq V \times C \leftarrow (\mathcal{F} \cap (V \times F_{lock})) \cup (\mathcal{B} \cap (V \times B_{lock}))$;
  $\overrightarrow{\mathcal{F}} \leftarrow \overrightarrow{\mathcal{F}_u} \cap \mathcal{C}on$;
  $\overrightarrow{\mathcal{B}} \leftarrow \overrightarrow{\mathcal{B}_u} \cap \mathcal{C}on$;
  **while** $\overrightarrow{\mathcal{F}} \neq \emptyset \wedge \overrightarrow{\mathcal{B}} \neq \emptyset$ **do**
    $\overrightarrow{\mathcal{F}} \leftarrow ($Post$(\mathfrak{G}, \overrightarrow{\mathcal{F}}) \cap \mathcal{C}on) \setminus \mathcal{F}$;
    $\overrightarrow{\mathcal{B}} \leftarrow ($Pre$(\mathfrak{G}, \overrightarrow{\mathcal{B}}) \cap \mathcal{C}on) \setminus \mathcal{B}$;
    $\mathcal{F} \leftarrow \mathcal{F} \cup \overrightarrow{\mathcal{F}}$;
    $\mathcal{B} \leftarrow \mathcal{B} \cup \overrightarrow{\mathcal{B}}$;
  **end**
  $\mathcal{W} \subseteq V \times C \leftarrow \mathcal{F} \cap \mathcal{B}$;
  $\mathfrak{R}_{scc} \leftarrow \mathfrak{R}_{scc} \cup$ Join($\mathcal{W}$);
  Decomposition($\mathfrak{G}, \mathfrak{R}_{scc}, \mathcal{V} \setminus \mathcal{C}on$);
  Decomposition($\mathfrak{G}, \mathfrak{R}_{scc}, \mathcal{C}on \setminus \mathcal{W}$);

**Function** Pivots($\mathcal{V}$)
  $\mathcal{P} \subseteq (V \times C) \leftarrow \emptyset$; $\mathcal{V}' \subseteq (V \times C) \leftarrow \mathcal{V}$;
  **while** $\mathcal{V}' \neq \emptyset$ **do**
    $(v, c) \leftarrow$ Pick($\mathcal{V}'$);
    $\mathcal{P} \leftarrow \mathcal{P} \cup (\{v\} \times \sigma_1(v, \mathcal{V}'))$;
    $\mathcal{V}' \leftarrow \mathcal{V}' \setminus (V \times$ Colours$(\mathcal{P}))$;
  **end**
  **return** $\mathcal{P}$; cannot simply operate with sets of graph vertices as the normal algorithm would. To that end, we use the notion of coloured vertex sets as introduced in Section 3.1 where the symbolic operations we perform on these sets have been described.

Initially, the algorithm starts with all vertices and colours, i.e. the full set $V \times C$. However, as the components are discovered, the intermediate results may contain different vertices appearing only for certain subsets of C. As a result, we often cannot pick a single pivot vertex that would be valid for all considered colours. Instead, we aim to pick a *pivot set* $P \subseteq V \times C$ such that for every colour that still appears in $\mathcal{V}$, the set contains *exactly* one vertex. Alternatively, one can also view the pivot set as a (partial) function from $C$ to $V$. This is done in the Pivots function.

The lock-step reachability procedure also cannot operate as in a standard graph. First of all, there can be colours where the forward reachability converges first, as well as colours where this happens for backward reachability. The algorithm thus has to account for both options simultaneously. Second, for each colour, the reachability can converge in a different number of steps. To deal with this problem, we introduce the $F_{lock}$ and $B_{lock}$ variables. These store the mutually disjoint sets of colours for which forward and backward reachability already converged. The lock-step procedure terminates when $F_{lock}$ and $B_{lock}$ contain all the colours that appear in $\mathcal{V}$.

Throughout the algorithm, we keep track of several coloured-set variables. The first two, $\mathcal{F}$ and $\mathcal{B}$, represent the forward and backward reachable sets, respectively. We then have four variables $\overrightarrow{\mathcal{F}}$, $\overrightarrow{\mathcal{F}_u}$, $\overrightarrow{\mathcal{B}}$, $\overrightarrow{\mathcal{B}_u}$ to represent the *frontiers* of these sets, i.e., the set of pairs $(v, c)$ such that the vertex $v$ has not yet been expanded in the corresponding reachability procedure for the colour $c$. The frontier of $\mathcal{F}$ is the set $\overrightarrow{\mathcal{F}} \cup \overrightarrow{\mathcal{F}_u}$. The sets $\overrightarrow{\mathcal{F}}$ and $\overrightarrow{\mathcal{F}_u}$ contain disjoint colours – $\overrightarrow{\mathcal{F}}$ involves those colours for which the lock-step reachability procedure has not finished yet, while $\overrightarrow{\mathcal{F}_u}$ represents the *unfinished* part of the frontier that shall be explored in the second while cycle; similarly for $\overrightarrow{\mathcal{B}}$ and $\overrightarrow{\mathcal{B}_u}$.

In the first while cycle (lines 10–21), we compute the reachability sets in the lock-step manner. Once a reachability set is completed for some colours (i.e., there are no vertices to expand with those colours), we add the colours to the corresponding $F_{lock}$ or $B_{lock}$ variable. Note that we ensure that $F_{lock}$ and $B_{lock}$ remain disjoint even if the two reachability procedures converged at the same time for certain colours—see line 14. We use $F_{lock}$ and $B_{lock}$ to split the newly computed frontier sets into the parts that are to be explored in the next iteration ($\overrightarrow{\mathcal{F}}$, $\overrightarrow{\mathcal{B}}$) and the parts that are currently left unfinished ($\overrightarrow{\mathcal{F}_u}$, $\overrightarrow{\mathcal{B}_u}$).

After the first while cycle, we compute the set $\mathcal{C}on$ that is an analogue for the converged set of the original lock-step algorithm (line 22). As already suggested above and unlike the original algorithm, this set cannot be just $\mathcal{F}$ or $\mathcal{B}$, but is instead a mixture of both, depending on the convergent colours. To compute this set, we use the $F_{lock}$ and $B_{lock}$ variables.

The second while cycle (lines 25–30) then completes the unfinished forward and backward reachability set, restricted to the inside of the converged set. The intersection of $\mathcal{F}$ and $\mathcal{B}$ then forms a coloured set $\mathcal{W}$ with the property that for all $c \in \text{Colours}(\mathcal{V})$, $\mathcal{W}(\_, c)$ is a strongly connected component of $\mathfrak{G}(c)$. We create the corresponding relation using the Join operation, add this relation to the resulting $\mathfrak{R}_{scc}$, and recursively call the whole procedure with $\mathcal{V} \setminus \mathcal{C}on$ and $\mathcal{C}on \setminus \mathcal{W}$ as the base coloured sets of vertices.

Let us note that there is possibly another approach. Instead of trying to work with all colours still appearing in the coloured vertex set at once, we cold fork a new recursive procedure whenever the colour set splits due to the differences in the graph structure. For example, instead of picking multiple coloured vertices as pivots, one could pick a single vertex with a valid subset of colours and then address the remaining colours in a separate recursive call. While such approach could be to some extent beneficial in a massively parallel environment where each recursive call can be executed independently on a new CPU, the amount of forking in large systems will soon become unreasonable. More importantly, it defeats the purpose of symbolic representation which aims to minimise the number of symbolic operations.

#### 3.5 Correctness and Complexity of the Coloured Lock-step Algorithm

**Theorem 1.** *Let $\mathfrak{G} = (V, C, E)$ be a coloured graph. The coloured lock-step algorithm terminates and computes the coloured SCC decomposition relation $\mathfrak{R}_{scc}$.*

*Proof.* We first show that the set $\mathcal{W}$ computed on line 31 indeed contains one SCC for every colour $c \in \text{Colours}(\mathcal{V})$ and that the recursive calls of Decomposition preserve the property that $\mathcal{V}$ is SCC-closed with respect to all colours.

Let us assume that $\mathcal{V}$ is SCC-closed and let us take an arbitrary $c \in \text{Colours}(\mathcal{V})$. The function Pivots chooses a set that contains exactly one pair whose colour is $c$, let us call this pair $(v, c)$. Let us further assume that $c$ is assigned into $F_{lock}$ first (the case with $B_{lock}$ is completely symmetric).

Let us now choose an arbitrary vertex $w$ such that $v$ and $w$ are in the same SCC of $\mathfrak{G}(c)$, i.e. $v \to^* w$ and $w \to^* v$. As the first while cycle finishes, $\mathcal{F}$ contains all the pairs of the form $(u, c) \in \mathcal{V}$ where $u$ is reachable from $v$ in $\mathfrak{G}(c)$. Thus, it also contains $(w, c)$ due to the fact that $\mathcal{V}$ is SCC-closed. Now, either $(w, c) \in \mathcal{B}$, or there exists a vertex $x$ such that $w \to^* x$, $x \to^* v$ in $\mathfrak{G}(c)$ and $x \in \overrightarrow{\mathcal{B}_u}$. This means that $(w, c)$ is added to $\mathcal{B}$ in the second while cycle. In both cases, both $(v, c)$ and $(w, c)$ are then added to $\mathcal{W}$. As the vertex choices were arbitrary, this proves that the SCC of $v$ in $\mathfrak{G}(c)$ is contained in $\mathcal{W}$. Furthermore, if $(y, c) \in \mathcal{W}$ for an arbitrary $y$, then $v \to^* y$ and $y \to^* v$ in $\mathfrak{G}(c)$, which means that $y$ is in $SCC(\mathfrak{G}(c), v)$. This proves that $\mathcal{W}$ contains exactly one SCC for every colour $c \in \text{Colours}(\mathcal{V})$.

We now argue that $\mathcal{C}on$ is SCC-closed with respect to all colours. This immediately implies that both $\mathcal{V} \setminus \mathcal{C}on$ and $\mathcal{C}on \setminus \mathcal{W}$ are SCC-closed. Let us assume that there is a colour $c \in \text{Colours}(\mathcal{V})$ and two vertices $v$, $w$ in the same SCC of $\mathfrak{G}(c)$ such that $(v, c) \in \mathcal{C}on$, but $(w, c) \notin \mathcal{C}on$. Let us assume that $c \in F_{lock}$ (as above, the case of $B_{lock}$ is completely symmetrical). Then $(v, c) \in \mathcal{F}$

after the first while cycle finishes. This also means that $(w, c) \in \mathcal{F}$ as the forward reachability procedure is completed for $c$ and thus $(w, c) \in \mathcal{C}on$, a contradiction.

What remains is to show that the algorithm terminates and that every SCC is eventually found. Termination is trivially proved by the fact that size of the set $\mathcal{V}$ always decreases in recursive calls: both $\mathcal{W}$ and $\mathcal{C}on$ are nonempty, because they contain the initial pivot set as a subset. Clearly, a representant of every SCC of every monochromatisation $\mathfrak{G}(c)$ is eventually chosen as a pivot. Together with the above reasoning, this implies that the algorithm is correct. □

**Theorem 2.** *Let $|V|$ be the number of vertices in the coloured graph and let $|C|$ be the number of colours. The coloured lock-step algorithm performs at most $\mathcal{O}(|C| \cdot |V| \cdot \log |V|)$ symbolic steps.*

*Proof.* Let us first note that all the derived operations defined in Fig. 1 use only a constant number of the basic symbolic operations. As we are considering asymptotic complexity here, we can view all the operations in Fig. 1 as elementary symbolic steps.

We first make the observation that each vertex may be chosen as a part of the pivot set at most $|C|$ times. Clearly, once a vertex is included in the pivot set with a set of colours $C'$, then, $\{v\} \times C' \subseteq \mathcal{C}on$ (due to the monotonicity of the construction of $\mathcal{F}$ and $\mathcal{B}$) and the elements of $\{v\} \times C'$ do not appear in subsequent recursive calls. This means that the total complexity of the calls to Pivots is bounded by $O(|C| \cdot |V|)$ and we can exclude the calls from the rest of the complexity analysis.

We now consider the complexity of a single call to Decomposition without the subsequent recursive calls. Let us now select one of the colours for which the lock-step reachability procedure (lines 10–21) finished last, i.e., one of the colours that have been added to $F_{lock}$ or $B_{lock}$ in the final iteration of the cycle. Let us call this colour $c$. Recall that $\sigma_2(c, \mathcal{X})$ is the set of vertices with colour $c$ in a coloured set $\mathcal{X}$.

Let us denote by $W := \sigma_2(c, \mathcal{W})$ and let $S$ be the smaller of $\sigma_2(c, \mathcal{V} \setminus \mathcal{C}on)$ and $\sigma_2(c, \mathcal{C}on \setminus \mathcal{W})$. Clearly $S$ contains at most $|V|/2$ vertices. Let $k = |S \cup W|$. We now argue that the number of symbolic steps in a given call (without the recursive calls) is bounded by $\mathcal{O}(k)$.

Assume w.l.o.g. that $c \in F_{lock}$ (a completely symmetric argument solves the case $c \in B_{lock}$). Then $\sigma_2(c, \mathcal{C}on) = \sigma_2(c, \mathcal{F})$. If $S$ is $\sigma_2(c, \mathcal{C}on \setminus \mathcal{W})$ then $k$ is the size of $\sigma_2(c, \mathcal{F})$. Each iteration of the first while cycle puts at least one vertex with colour $c$ into $\mathcal{F}$; otherwise $c$ would not be one of the last colours to finish. This means that the cycle runs for at most $k$ iterations. This also means that the size of $\sigma_2(x, \mathcal{X})$ for all colours $x$ and $\mathcal{X} \in \{\mathcal{F}, \mathcal{B}\}$ is also bound by $k$, which in turn means that the second while cycle cannot make more than $O(k)$ steps.

If $S$ is $\sigma_2(c, \mathcal{V} \setminus \mathcal{C}on)$ instead, let us define $B := \sigma_2(c, \mathcal{B})$ right after the first while cycle has finished. We know that $B \subseteq S \cup W$: if a vertex $v$ were in $B \setminus S$ then $(v, c) \in \mathcal{C}on = \mathcal{F}$ and thus $v \in W$. Again, each iteration of the first while cycle puts at least one vertex with colour $c$ into $\mathcal{B}$; otherwise $c$ would have been in $B_{lock}$ before it appeared in $F_{lock}$. Similarly to the previous case, this means that both while cycles run for at most $O(k)$ steps.

The rest of the argument uses amortised reasoning, in a way similar to the proof in [14]. Note that each vertex is going to be an element of the set $W$ as described above at most $|C|$ times (once for each colour). Furthermore, each vertex is going to be an element of the set $S$ as described above at most $|C| \cdot \log |V|$ times: for each colour, the vertex can be an element of the smaller of the two sets at most $\log |V|$ times. As the cost of each single call can be charged to the vertices in $S \cup W$ as explained above, it is sufficient to charge each vertex the total cost of $|C| + |C| \cdot \log |V|$. Together, this means that the total number of symbolic steps is bounded by $O(|C| \cdot |V| \cdot \log |V|)$. □

Note that the upper bound established by Theorem 2 is no better than the one we would get if we split the coloured graph into its monochromatic constituents and processed each monochromatic graph separately using the original lock-step algorithm [14]. We remark, however, that the coloured approach is a heuristic whose real complexity might be much smaller. Indeed, the complexity analysis in the previous proof focused on a single colour, omitting the fact than SCCs for many other colours are found at the same time. In case where the edges are largely shared among the colours, which is true in many applications, the heuristic has the potential to significantly outperform the parameter-scan approach. The situation is similar to that of the coloured model checking; see the observations made in [5].

## 4 Experimental Evaluation

In this section, we examine the applicability of our algorithm in real-world situations. First, we discuss how we implemented the algorithm and share some useful recommendations in this regard. We then look at how the implementation performs on real-life coloured graphs which are derived from large models considered in computational biology.

### 4.1 Implementation

As our symbolic set representation, we consider standard reduced ordered binary decision diagrams (ROBDDs, or just BDDs for short) [17]. The source of our edge-coloured graphs are the transition systems of *parametrised Boolean networks* (PBN) as understood in [11, 60].

**Boolean networks.** Normal (non-parametrised) Boolean networks [34, 46, 49, 54] appear in computational systems biology as logical models of complex biochemical processes [16]. Here, we use the asynchronous variant of BNs introduced by Thomas [54]. A Boolean network consists of Boolean variables, each having a Boolean update function. Update functions are executed non-deterministically and change the state of the Boolean variables. The semantics of such a network is a directed graph where the vertices are the possible valuations of the Boolean variables and the edges are induced by the non-deterministic execution of the update functions.

This type of models is especially challenging for symbolic analysis. It is a well-known fact, that using symbolic structures, like BDDs, to represent very large state spaces gives good results for synchronous systems, but shows its limits when trying to tackle asynchronicity (see e.g. [23]).

In the parametrised variant, the update functions can be partially unknown. This introduces a set of colours (parametrisations), each colour fully instantiating all update functions of the network. As a result, the semantics of such a model is an edge-coloured directed graph as we consider in this paper. For a full technical description of PBNs and their coloured graph semantics, please refer to [11].

Our implementation heavily relies on the existing internal libraries of our tool AEON [12], which at the moment partially supports symbolic analysis of PBNs. Specifically, AEON uses symbolic BDD-based representation of colour sets, but relies on explicit state space exploration. In this work, we extend these capabilities to fully symbolic analysis of the whole graph.

**Custom operations.** Aside from implementing the Post and Pre operations for a given PBN, we also choose to provide specialised implementations for the Colours and Pivots procedures. Especially for the Pivots procedure, this can greatly reduce the number of necessary symbolic steps, as we avoid picking pivots vertex-by-vertex.

To implement these two operations as efficiently as possible, we always order the Boolean variables in our BDDs starting from the colour and ending with vertex variables. This ensures that both Pivots and Colours can be implemented by pruning the vertex variable nodes and minimising the BDD.

Specifically, in this ordering, for Colours, all vertex nodes are effectively substituted with the `true` terminal node and the BDD is minimised. For Pivots, one (arbitrary) path of vertex variable nodes (corresponding to one pivot vertex) is preserved for every colour, and the rest of the vertex nodes are pruned.

**Trimming.** Finally, most graphs typically contain a large number of trivial SCCs that introduce unnecessary overhead to the main algorithm. To avoid this overhead, we additionally perform a trimming step before each invocation of Decomposition. Trimming consists of repeatedly removing all vertices which have no outgoing or no incoming edges and is employed by most symbolic SCC algorithms on standard directed graphs as well. The coloured analogue of trimming is straightforward, as it can be achieved using Pre and Post operations just as in the non-coloured case. For a coloured set of vertices $\mathcal{V}$, $\textsc{Post}(\textsc{Pre}(\mathfrak{G}, \mathcal{V}) \cap \mathcal{V}) \cap \mathcal{V}$ returns only vertices which have at least one predecessor in $\mathcal{V}$. The successor variant simply exchanges the Post and Pre operations.

### 4.2 Experiments

We evaluated our algorithm on 7 real-world networks based on the models from the Ginsim Boolean network database [19]. The experiments were performed on a 32-core AMD Ryzen workstation with 64GB of RAM memory. All tested models are available in our source code repository.[3] Note that the smaller models

[3] `https://github.com/sybila/biodivine-lib-param-bn/tree/tacas`

**Table 1.** Overview of the test models for the algorithm evaluation. The times (`minutes:seconds`) refer to the total runtime of the SCC decomposition procedure. The model *variables* and *parameters* give the number of Boolean variables necessary to represent the PBN symbolically. Finally, the *graph size* and *colour set size* specifies the magnitude of $|V| \cdot |C|$ and $|C|$ for the coloured graph corresponding to the network.

| Model Name | Model Variables | Model Parameters | Graph Size | Colour Set Size | Time |
|---|---|---|---|---|---|
| Asymmetric Cell Division [48] | 5 | 48 | $\sim 2^{24}$ | $\sim 2^{19}$ | 00:09.47 |
| Reduced TCR Signalisation [36] | 10 | 46 | $\sim 2^{24}$ | $\sim 2^{14}$ | 00:58.35 |
| Budding Yeast (Orlando) [43] | 9 | 54 | $\sim 2^{27}$ | $\sim 2^{18}$ | 01:13.39 |
| Budding Yeast (Irons) [31] | 18 | 44 | $\sim 2^{35}$ | $\sim 2^{17}$ | 50:44.80 |
| T-Cell Differentiation [41] | 23 | 48 | $\sim 2^{40}$ | $\sim 2^{17}$ | 71:80.12 |
| WG Signalling Pathway [40] | 26 | 38 | $\sim 2^{48}$ | $\sim 2^{22}$ | 78:38.34 |
| Full TCR Signalisation [36] | 30 | 48 | $\sim 2^{47}$ | $\sim 2^{17}$ | 118:34.88 |

($< 2^{30}$) should be easy to process even on a less powerful machine, however the larger models can require substantial amounts of RAM.

The PBNs and their analysis runtime is summarised in Table 1. For each network, we specify the number of Boolean variables used by symbolic encoding, separated into model variables (vertices) and model parameters (colours), and the actual approximate size of the coloured graph. Note that not all combinations of parameters (possible graph colours) are usually biologically admissible, and these are filtered out before the coloured SCC decomposition. Hence the size of the graph is smaller than the space of all the considered BDD variables.

From the presented results, we can draw the following observations: First, fully symbolic approach allows us to scale to much larger graphs than before, especially in terms of state space. Until now, AEON was typically limited (even for an easier problem of bottom SCC detection) to vertex counts of $2^{15} - 2^{20}$, exhausting memory even for much smaller state spaces when dealing with complex parameter space. Here, we can easily handle up to $2^{30}$ vertices with non-trivial parameter space and we hope to push this number even higher with further optimisations to our experimental implementation.

Second, the coloured heuristic is beneficial for symbolic computation. To support this claim, we considered a monochromatic variant of the decomposition problem for the WG Signaling Pathway and tested the basic lock-step algorithm on a collection of pseudo-random monochromatisations of this graph. Processing one such monochromatisation typically required $0.5 - 1$ second. Considering the graph in question has 2359296 colours, processing the colours one-by-one would, even in ideal conditions, take well above 300 hours (more than 12 days).

### 5 Conclusions

In this paper we have presented a fully symbolic algorithm for detecting all monochromatic strongly connected components in edge-coloured graphs. The work has been motivated by systems sciences, namely systems biology, where the need for efficient automated analysis of components in large graphs with large sets of coloured edges is emergent. The algorithm combines several ideas inspired by existing state-of-the-art algorithms for SCC decomposition in a non-trivial way. We believe this is the first fully symbolic algorithm aiming to solve the problem efficiently.

The experimental evaluation has shown that in expected practical scenarios, the presented algorithm has a strong potential to be significantly faster than iterating a standard algorithm for SCC decomposition executed on all monochromatic sub-graphs one-by-one.
